# Pulse intensity characterization of the LCLS nanosecond double-bunch mode of operation

**DOI:** 10.1107/S160057751800348X

**Published:** 2018-03-27

**Authors:** Yanwen Sun, Franz-Josef Decker, James Turner, Sanghoon Song, Aymeric Robert, Diling Zhu

**Affiliations:** a Linac Coherent Light Source, SLAC National Accelerator Laboratory, Menlo Park, CA 94025, USA; b Physics Department, Stanford University, CA 94305, USA

**Keywords:** FEL, X-ray, XSVS, speckle, XPCS

## Abstract

The recent demonstration of the ‘nanosecond double-bunch’ operation mode, *i.e.* two X-ray pulses separated in time between 0.35 and hundreds of nanoseconds and by increments of 0.35 ns, offers new opportunities to investigate ultrafast dynamics in diverse systems of interest. However, in order to reach its full potential, this mode of operation requires the precise characterization of the intensity of each X-ray pulse within each pulse pair for any time separation. Here, a transmissive single-shot diagnostic that achieves this goal for time separations larger than 0.7 ns with a precision better than 5% is presented. It also provides real-time monitoring feedback to help tune the accelerator parameters to deliver double pulse intensity distributions optimized for specific experimental goals.

## Abstract 2

Details and performances of a transmissive pulse intensity diagnostic for the LCLS ‘nanosecond double-bunch’ mode are presented.

## 1. Introduction   

Free electron lasers (FELs) provide very short, very intense and nearly fully transversely coherent pulses of X-rays with typically >10^11^ coherent photons contained in a pulse of duration below 100 fs. These new sources have already revealed some of their potential by revolutionizing various experimental approaches using, for example, ‘diffract-before-destroy’ in combination or not with pump–probe experimental approaches. Therefore, X-ray FELs offer unique opportunities to probe structural and dynamical information in many systems of interest in fields as diverse as life sciences, chemistry and material sciences, to name a few (Bostedt *et al.*, 2016 ▸). The FEL X-ray pulses’ unique properties, in combination with the possibility of generating two such pulses with a controlled temporal separation in the femtosecond to nanosecond timescales, are opening up new opportunities for the investigation of equilibrium and non-equilibrium dynamics in a broad range of systems. X-ray pump/X-ray probe experiments could, for example, lead to a better understanding of X-ray induced dynamics and provide more information on the fundamentals of X-ray/matter interactions at high pulse intensities (Vinko *et al.*, 2012 ▸; David *et al.*, 2015 ▸; Briggs *et al.*, 2017 ▸). This can also be applied to dramatically extend to much shorter timescales X-ray photon correlation spectroscopy (XPCS) studies (Grübel *et al.*, 2008 ▸) by means of X-ray speckle visibility spectroscopy (XSVS).

XPCS, as routinely performed with synchrotron light sources, probes dynamics on timescales from milliseconds to seconds or longer (Grübel *et al.*, 2008 ▸). It relies on measuring time-resolved coherent scattering patterns, *i.e.* speckle patterns. This technique is mostly limited at synchrotron sources by the available coherent flux and/or, in many cases, by the performance of suitable X-ray imaging detectors (*i.e.* frame rate, pixel size, noise, single-photon sensitivity, *etc.*). Today’s state-of-the-art imaging detectors suitable for XPCS typically operate at about 1 kHz, which therefore allows dynamics to be routinely probed down to the millisecond. With the development of diffraction-limited storage ring sources (Eriksson *et al.*, 2014 ▸), the availability of much larger coherent flux together with the development of faster detectors will allow XPCS studies to extend to much faster timescales, which in turn will mostly be limited by the frame rate of the detector. Any increase of the frame rate of these detectors will allow faster dynamics to be measured than previously available, *i.e.* possibly down to the microsecond timescale. X-ray FELs, which are pulsed sources by nature, take direct advantage of the large and nearly fully transversely coherent flux of each X-ray pulse. They, therefore, offer the possibility to perform XPCS studies for probing timescales longer than their intrinsic repetition rate, assuming a suitable detector records a speckle pattern for every FEL pulse (Carnis *et al.*, 2014 ▸; Lehmkühler *et al.*, 2015 ▸).

In order to access faster timescales, other schemes not relying solely on the existing performances of detectors in terms of frame rate can be used. XSVS is, for example, based on its analog in the visible range (Dixon & Durian, 2003 ▸; Bandyopadhyay *et al.*, 2005 ▸). On storage rings, it relies on analyzing the speckle visibility or contrast as a function of the exposure time, with the advantage that it can access dynamics faster than the repetition rate of the detector (Inoue *et al.*, 2012 ▸; DeCaro *et al.*, 2013 ▸; Li *et al.*, 2014 ▸). At FELs, all X-ray photons are delivered to the sample in a very short time (<100 fs) and the current state-of-the-art detectors have an integration time orders of magnitude larger than the X-ray pulse duration. XSVS at FELs can, however, similarly be performed by varying the X-ray pulse duration 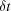 instead of the acquisition time and is then only limited by the accessible range of 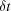 and the ability to tune within this range. Such a scheme could potentially offer the possibility to measure dynamics with a timescale of the order of several femto­seconds.

Two-pulse XSVS (2P-XSVS) is another promising approach that relies on the generation of two identical X-ray pulses with a time separation 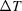 shorter than the integration time of the detector. It is then limited by the ability to tune 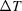 (Gutt *et al.*, 2009 ▸) and has recently been supported by the developments of X-ray split-and-delays. These complex X-ray optics generically rely on the same principles: each pulse is first split into two X-ray beams by an X-ray optical element; the two X-ray pulses then propagate on paths of different lengths before being recombined onto a single trajectory directed to the sample.

Various efforts have been and are being pursued that result in the possibility of providing 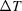 ranging from zero up to typically a fraction or a couple of nanoseconds (Roseker *et al.*, 2009 ▸, 2011 ▸; David *et al.*, 2015 ▸; Osaka *et al.*, 2016 ▸, 2017 ▸; Sakamoto *et al.*, 2017 ▸; Zhu *et al.*, 2017 ▸; Hirano *et al.*, 2018 ▸). Such time separations correspond to a path-length difference of typically 1 m. An extension to even larger delays seems therefore not straightforward to achieve with X-ray split-and-delays. Similarly, providing very short time separations below tens of femtoseconds with a high accuracy (*i.e.* 1 fs corresponding to a distance of ∼300 nm) would require an exquisite control of the path-length difference. In order to circumvent this, alternative accelerator-based schemes have been demonstrated at the Linac Coherent Light Source (LCLS) to produce two X-ray pulses separated in time with independently controllable properties. This is in particular highlighted by the following examples: (i) double-slotted foil (Emma *et al.*, 2004 ▸); (ii) hard X-ray two-color self-seeding (Lutman *et al.*, 2014 ▸; Marinelli *et al.*, 2015 ▸); (iii) two-color soft X-rays (Lutman *et al.*, 2013 ▸); (iv) nanosecond double-bunch (Decker *et al.*, 2010 ▸). They all rely to first order on the self-amplified spontaneous emission (SASE) process. Therefore inherent fluctuations are not only observed in the X-ray beam characteristics between pulse pairs but also between each pulse within a pulse pair (Bonifacio *et al.*, 1994 ▸). These fluctuations can influence many characteristics of each X-ray pulse such as timing, pulse intensity, pointing or even photon energy. They also complicate the interpretation of experimental data, which can be circumvented if diagnostics characterize appropriately each individual pulse within a pulse pair.

We present here the details and performances of a non-destructive pulse intensity diagnostic that provides that capability for the hard X-ray nanosecond double-bunch mode. It utilizes a high-speed photodetector with a sufficiently fast response to measure the time-resolved signal of the two pulses within each pulse pair. This diagnostic is also revealed to be essential as an online monitoring feedback to tailor the fine details of the X-ray characteristics of each pulse pair by tuning the FEL performance.

## 2. Experimental setup   

In the following we describe the details and performances of a diagnostic that allows the pulse intensity of each X-ray pulse within a pulse pair generated in the so-called ‘nanosecond double-bunch’ operation mode to be measured.

This mode of operation relies on using two independent injector laser pulses, which are both synchronized to the master radiofrequency (RF) of the accelerator. Both injector laser pulses strike the photocathode with a time separation 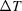 which is precisely a multiple of the accelerating field, *i.e.* a so-called ‘RF bucket’. For LCLS this corresponds to an incremental separation time of 0.35 ns and can provide separation times up to several hundreds of nanoseconds. The two electron bunches are then accelerated to their nominal energy and introduced in the undulator. They both emit X-rays independently *via* the SASE process (Decker *et al.*, 2015 ▸). Whereas both electron bunches experience some level of common fluctuations while accelerated through the linear accelerator, because of the inherent stochastic nature of the SASE process itself, many X-ray beam properties for each pulse will present differences. This is, in particular, the case for the pulse intensity of each pulse within a pulse pair, but can also affect at the pulse level the precise photon energy, spatial profile, *etc*.

These differences can be critical for the data analysis and interpretation of some experiments. 2P-XSVS experiments, for example, require that, in addition to being spatially overlapped, each pulse within a pulse pair is as identical as possible, *i.e.* with the same X-ray photon energy and bandwidth and a well characterized pulse intensity ratio between the first and second pulse (Gutt *et al.*, 2009 ▸; Sun *et al.*, 2017 ▸). Other applications such as X-ray pump/X-ray probe experiments may typically require that the first pulse (pump) is stronger than the second one (probe). It can also be advantageous in some cases to have significant differences in photon energy.

A rough estimate of the pulse intensities within each pulse pair can already be provided from the X-band transverse cavity (XTCAV) diagnostic (Ding *et al.*, 2011 ▸). It has been revealed, however, to be computationally demanding and can only provide the relative pulse intensity ratio with limited sensitivity and accuracy. More importantly, the XTCAV can provide an estimate of the relative pulse intensities but only immediately downstream of the undulator. However, some experiments, such as 2P-XSVS, require the two pulses to have an increased and controlled longitudinal coherence, which is performed by using a monochromator. The XTCAV can then no longer provide a representation of the pulse intensity ratio between the pulses within each pulse pair.

We therefore developed and characterized a pulse intensity diagnostic that can provide the critical information to perform 2P-XSVS experiments. It was installed at the X-ray correlation spectroscopy instrument (XCS) at LCLS (Alonso-Mori *et al.*, 2015 ▸). The pulse pairs were monochromatized at 8.2 keV using a Si(111) K-monochromator located in the front-end enclosure (Welch *et al.*, 2009 ▸). The beam is then offset from the undulator axis by a pair of X-ray mirrors (Moeller *et al.*, 2011 ▸). Both X-ray pulses within each pair are focused to the sample location by using beryllium compound refractive lenses with a focal length of ∼3.3 m.

**Figure 1:**
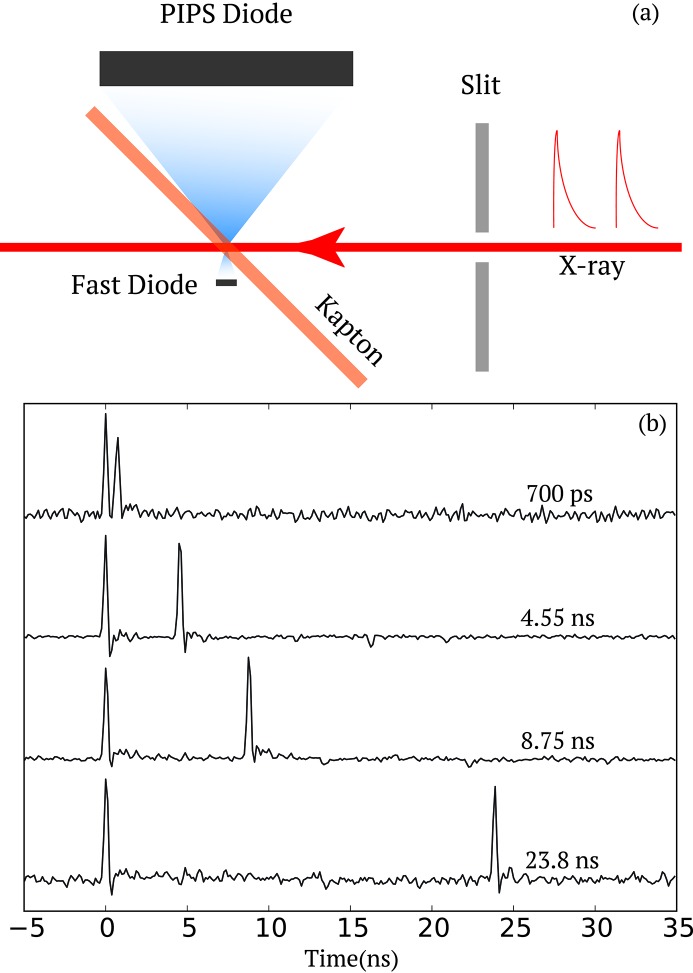
(*a*) Pulse intensity diagnostic consisting of a fast diode (active area 0.2 mm × 0.2 mm) and a PIPS diode (active area 300 mm^2^). Both diodes measure the scattering from a thin (150 µm) Kapton film. A pair of slits is located upstream to reduce the spatial extent of the beam if needed. (*b*) Examples of single-shot raw traces from the Acqiris digitizer for two pulses at various time separations 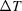 of 0.70, 4.55, 8.75 and 23.80 ns.

This diagnostic consists of a Hamamatsu GaAs metal–semiconductor–metal (MSM) photodetector (Sato *et al.*, 2013 ▸) that collects scattered photons from a 150 µm-thick Kapton film, installed at ∼45° incidence angle, as illustrated in Fig. 1(*a*) ▸. The Kapton thickness allows most of the X-rays to reach the samples with negligible absorption losses (∼15% at 8.2 keV). A 9 V bias was applied to the MSM detector. The signal is further amplified using two 10 dB broadband voltage amplifiers (Mini-Circuits ZX60-14012L-S+) before being read out with an Acqiris digitizer at 8 GHz. Concurrently a PIPS (passivated implanted planar silicon; Canberra PIPS FD300) diode was mounted on the other side of the Kapton film to collect the scattered photons as a reference measurement. The PIPS diode has a much larger collection area, albeit much slower response time, so that it measures the integrated pulse intensity of both pulses within a pair. The Kapton–MSM–PIPS assembly was mounted on a two-axis translation stage to optimize the sensor and Kapton film positions in order to achieve the best measurement performance. The active area of the MSM is 0.2 mm × 0.2 mm. We found that the optimum distance between where the X-ray beam hits the Kapton film and the MSM sensor surface is between 400 and 800 µm. In the Far Experimental Hall, the unfocused incident beam size at the XCS instrument is about 750 µm (Alonso-Mori *et al.*, 2015 ▸). A pair of 200 µm slits located 0.6 m from the sample (*i.e.* 0.3 m from the fast diode) was used to not only minimize the parasitic small-angle scatterings around the beam but also the nearly unfocused third-harmonic content that could hit the MSM directly and produce unwanted background.

In Fig. 1(*b*) ▸, a series of single-shot two-pulse time traces are displayed for various time separations 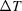 ranging from 0.7 to 23.8 ns. They were selected from the many measurements to represent pulse pairs where the pulse intensity of the first and second pulse are similar. It unambiguously demonstrates that the two pulses can be properly resolved, down to a time separation of 0.7 ns (*i.e.* corresponding to two RF buckets). When the two pulses are separated by only a single RF bucket (*i.e.* 0.35 ns), the signals cannot be disentangled (data not shown). The time resolution of the diagnostic, in this case, is limited by the 8 GHz sampling rate and 2 GHz bandpass filter of the digitizer.

For a given time separation 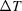 we recorded many traces of the pulse intensity measured by the MSM diode. The pulse intensity of each individual pulse is obtained by integrating the time trace 250 ps before the peak position and 375 ps after. The integration range has been used as an optimization parameter for the performance of the diagnostic in terms of precision as described later. An offset (corresponding to the integrated signal over 12.5 ns in the baseline before the first peak) is subtracted for each pulse. This implies that the minimum time separation between the two pulses must be longer than five sampling intervals, thus corresponding to 625 ps. This confirms that the diagnostic performances can only be ensured for time separations between pulses corresponding to two RF buckets, thus translating into 700 ps.

## 3. Discussion   

In the following sections we described some of the relevant X-ray beam parameters for each pulse within a pulse pair primarily for the purpose of performing 2P-XSVS experiments. This could be of interest for other scientific applications with different requirements. A detailed analysis of the overall behavior of these pulse pairs is provided in terms of: (i) pulse intensity measurement precision, (ii) pulse intensity jitter and (iii) electron energy jitter.

### 3.1. Precision on the pulse intensity measurement   

**Figure 2:**
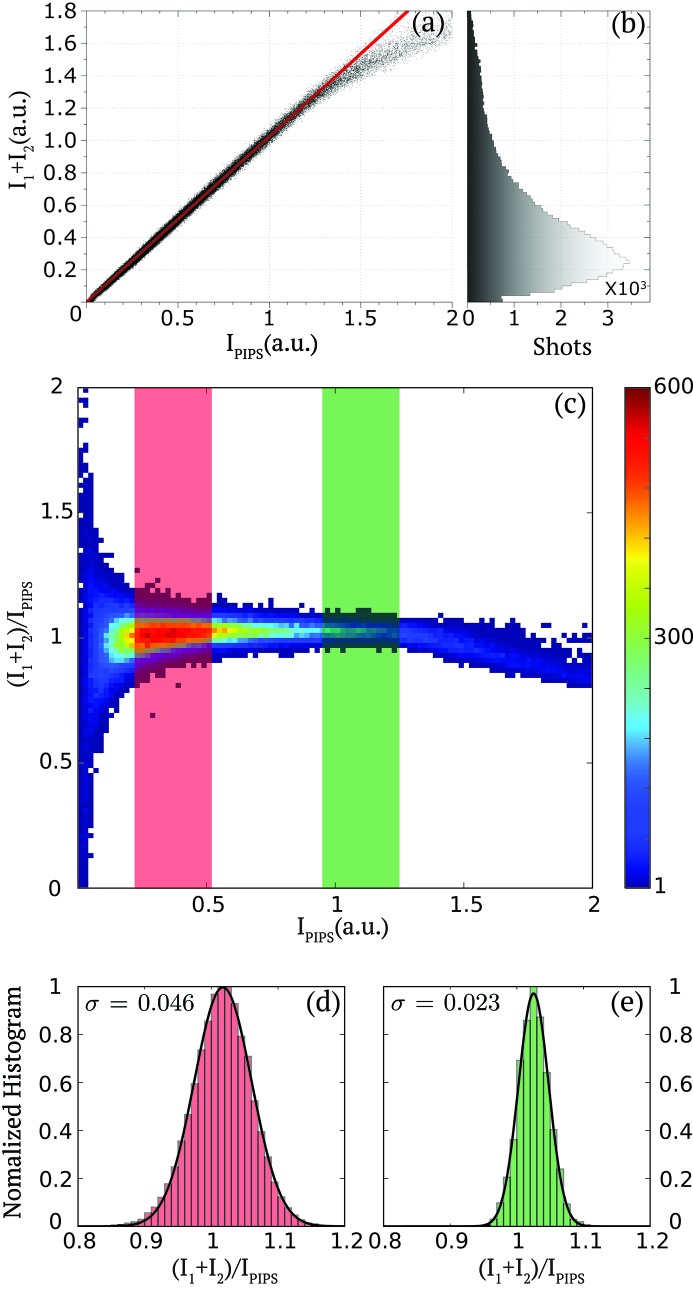
(*a*) Pulse intensity correlation between the fast diode measurement of the summed-pulse intensities in each pulse (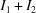) and the PIPS diode signal 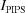 for a time separation 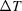 = 8.75 ns. (*b*) Histogram of the summed-pulse intensities (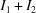) distribution measured by the fast diode from 10^5^ shots. (*c*) Two-dimensional cumulative histogram of the pulse intensity ratio of fast diode summed-pulse intensity and PIPS diode 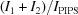 as a function of the PIPS diode signal 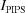 from 10^5^ shots. The color bar indicates counts. (*d*, *e*) Histograms and Gaussian fits (solid line) of the PIPS normalized pulse intensity of the pulse pairs 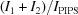 for the two signal levels of 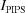 indicated by the red and green areas in (*c*).

In order to quantify the precision of the pulse intensity measurements of each pulse pair by the diagnostic, we display in Fig. 2(*a*) ▸ the correlation between the sum of the measured pulse intensities of the two pulses by the diagnostic 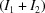 with that from the PIPS diode 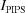. The PIPS diode measures the integrated signal of both pulses and is known to have a good linearity (Gabrysch *et al.*, 2008 ▸). The diagnostic shows a good linearity over its entire range, as highlighted with the solid line as a guide to the eye, with a slight saturation for shots where both pulses present large pulse intensities corresponding to 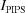 > 1.25 a.u. The saturated shots are, however, rare statistical events, as illustrated in Fig. 2(*b*) ▸, which represents the distribution of the total pulse intensity 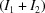 of many (10^5^) shots. The distribution clearly indicates that the vast majority of the pulse pairs consist of two pulses with a summed signal of moderate pulse intensity. We therefore optimized the distance between the MSM and the Kapton film in order to ensure that the dynamic range of our measurement covers the vast majority of the double pulse-pair pulse intensities. The small fraction of the shots presenting a pulse pair with a pulse intensity showing some saturation (*i.e.* <3%) can be further corrected by using an appropriate nonlinear response model if available or alternatively be clearly identified and further discarded in the data analysis.

In order to quantitatively estimate the uncertainty of the measurements with the fast diode, we plot the two-dimensional cumulative histogram of the summed-pulse intensities measured by the fast diode normalized by the PIPS diode signal 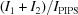 as a function of 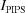. The vertical spread of the distribution indicates to what extent both signals are correlated. As an example we consider two representative levels of signals: (i) [0.22; 0.52], as indicated by the red area, which corresponds to the condition of most pulse pairs; and (ii) [0.95; 1.25], as indicated by the green area, which is a regime corresponding to strong summed-pulse intensities and just before any saturation effects are observed. The signal levels where 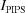 > 1.25 clearly present some saturation as indicated by the non-centrosymmetry of the distribution around unity. To further describe the precision of the measurement, Figs. 2(*d*) and 2(*e*) ▸ display the histogram of 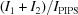 for pulse pairs corresponding, respectively, to the signal levels (i) and (ii) described previously. Each distribution was fitted by a Gaussian distribution, as indicated by the solid line, which leads to a standard deviation 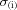 = 0.046 and 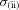 = 0.023 for the areas (i) and (ii), respectively. This confirms that for the vast majority of the pulse pair (and with a Kapton thickness of 150 µm) the precision of the measurement is better than 5%.

This diagnostic can obviously be used for detecting the signal of each double pulse pair under various beam conditions: different X-ray photon energies, different monochromaticity, *etc*. The pink beam signal would, for example, be typically about a factor of 50–100 stronger than under the present monochromatic conditions. The fast diode signal would therefore have to be optimized by determining an acceptable trade-off between Kapton thickness, scattered signal and diode-to-Kapton distance. This would be achieved by maximizing accuracy and optimizing the dynamic range while minimizing the fraction of saturated shots.

### 3.2. Pulse intensity fluctuations   

In the following, we characterize the pulse intensity distributions between the pulse pairs. The details of the relative pulse intensity distributions and correlations between each pulse within a pulse pair are of critical importance. This is especially the case for applications which require a specific combination of pulse pair intensities, as defined by the scientific needs of a given experiment. In the case of 2P-XSVS experiments, in the ideal case, each pulse intensity within a pair is preferred to be equal. Otherwise, the relative pulse intensity ratio between the two pulses is required to be measured with a certain precision in order to analyze, correct and further bin the data. For an X-ray pump/X-ray probe experiment one may, however, prefer to have the first pulse intensity larger than the second one in order to effectively ensure that a significant fraction of the shots are pumping the sample before probing it.

**Figure 3:**
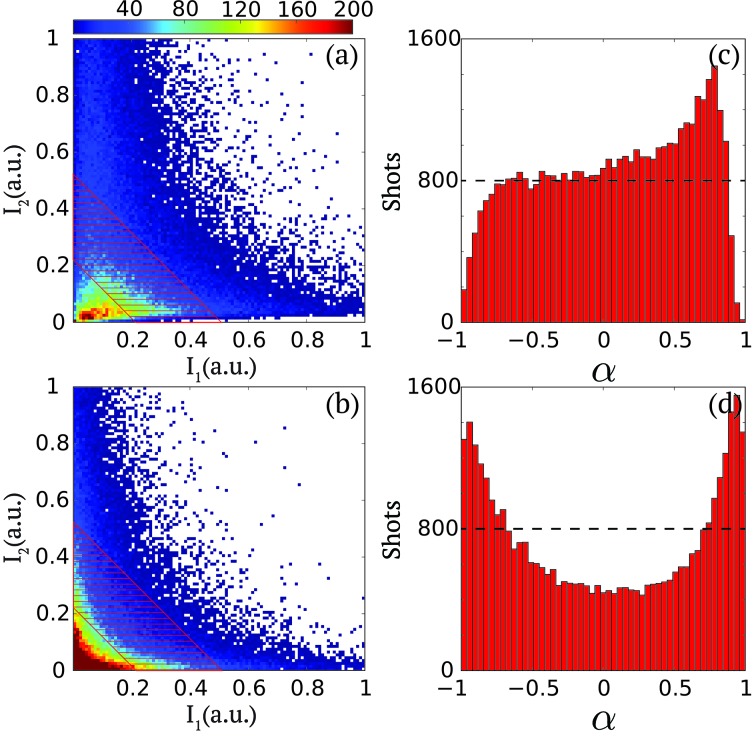
(*a*, *b*) Two-dimensional cumulative histograms of 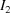 *versus* 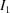 for two different operation configurations from 10^5^ shots. The color bar indicates counts. (*c*, *d*) Corresponding distributions of pulse intensity asymmetry 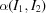 = 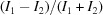 within the summed-pulse intensity regime as approximately illustrated by the dashed area, which corresponds to the 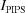 signal level within the [0.22, 0.52] range as indicated by the red area in Fig. 2(*c*) ▸.

These very different configurations can be favored to some extent by tuning the linac configuration, as highlighted in Figs. 3(*a*) and 3(*b*) ▸. They display the cumulative histograms of the pulse intensities measured in the first (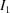) and the second (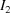) pulse in a series of 10^5^ pulses for two different configurations. In configuration (1), represented in Fig. 3(*a*) ▸, the pulse intensity distributions of both pulses are scattered in the lower diagonal of the histogram with most likely values of pulse intensity pairs located in the [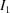, 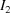] = [0.3, 0.2] area. In configuration (2), represented in Fig. 3(*b*) ▸, one can notice that the most likely pulse intensity pairs consist of shots where either 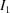 or 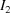 are strong. It also corresponds to an overall reduction of the pulse pair variety of pulse intensity configurations [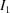, 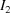]. There is especially a reduction of the number of shots where 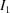 ≃ 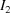, which as described previously would be the preferred two-pulse configuration for 2P-XSVS applications.

In order to quantitatively characterize these behaviors, we introduce a pulse intensity asymmetry parameter 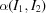 as
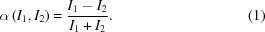
The asymmetry parameter α varies in the range [−1, 1] and presents the following distinct features, 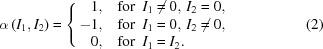
The histograms of α are plotted in Figs. 3(*c*) and 3(*d*) ▸ for the two previous configurations described in Figs. 3(*a*) and 3(*b*) ▸, respectively. They are specifically calculated for a subset of the data points approximately contained within the dashed areas in Figs. 3(*a*) and 3(*b*) ▸, which correspond to the 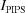 signal levels contained in the [0.22, 0.52] range as indicated by the red area in Fig. 2(*c*) ▸. One observes drastic differences between the two histograms. For configuration (1), the likelihood of having shots where only one of the two pulses contains some intensity is almost zero (*i.e.* those corresponding to 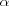 = 1 or −1). There is, however, a broad selection of shots where both the first and second pulses contain pulse intensity. As mentioned previously, the possibility of measuring for each shot the relative pulse intensity ensures that all shots could be used in the data analysis. For configuration (2), one observes that in contrast to configuration (1) the vast majority of the shots consist of pairs where the pulse intensity is mostly contained in either of the two pulses and not in both. Shots that contain pulses of equal pulse intensity are, for example, reduced by a factor of two. This indicates that, for 2P-XSVS experiments, configuration (1) would be more adequate whereas, for X-ray pump/X-ray probe experiments, configuration (2) would be more appropriate.

### 3.3. Electron energy jitter   

In order to further elucidate possible mechanisms behind the very different distributions shown in Fig. 3 ▸, we investigate the correlations between the pulse intensity of the first and second pulse downstream of the monochromator and the measured electron beam energies.

For each shot, either containing two pulses or not, the mean electron beam energy 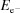 slightly differs due to various factors in the RF system, which results in an electron energy jitter distribution. In the case of two pulses per shot, the diagnostics providing 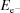 measures the average of 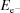 from the first and second pulse.

**Figure 4:**
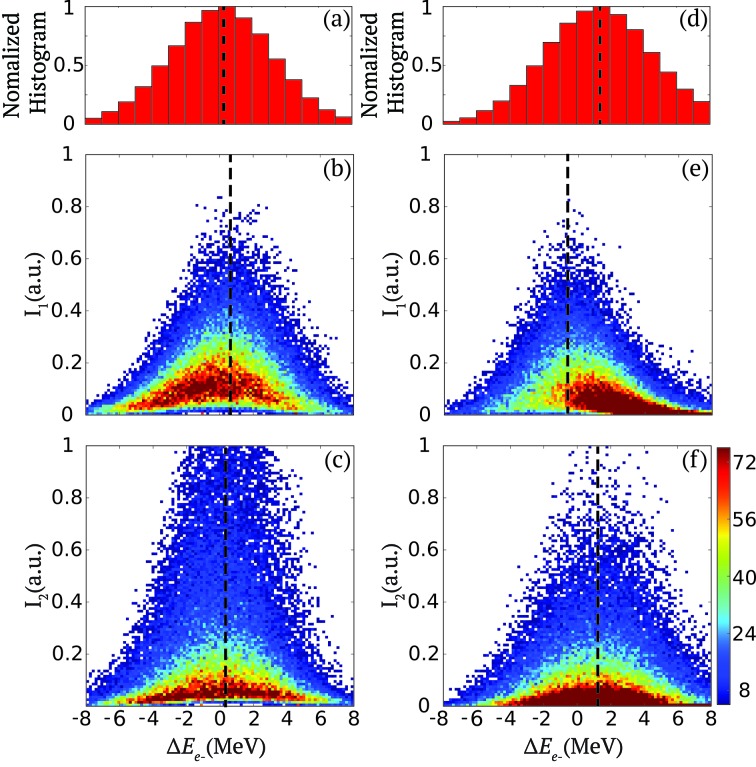
Configuration (1): (*a*) Histogram of the reduced electron beam energy 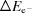 = 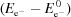 where 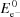 = 13478 MeV. (*b*, *c*) Two-dimensional cumulative histograms of the first and second pulse intensity 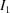 and 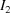 as a function of the the reduced electron beam energy 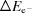 from 10^5^ shots. Configuration (2): (*d*, *e*, *f*) The same quantities as given in (*a*, *b*, *c*), respectively, for this configuration. The color bar indicates counts and the dashed lines the averaged of the distributions.

This is, for example, illustrated in Figs. 4(*a*) and 4(*d*) ▸ which show the normalized histogram of the reduced electron energy 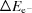 = 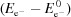, where 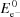 = 13478 MeV, when two pulses are present for each shot for the two configurations (1) and (2), respectively. The two distributions are very similar despite the very different behavior of 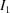, 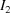 and 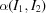 described previously.

The electron energy jitter translates into fluctuations of the centroid of the SASE spectrum around a central energy with respect to the X-ray energy band-pass set by the K-monochromator. Therefore, monochromatic pulses of large intensity typically correspond to cases where the centroid of the SASE spectrum coincides with the central energy of the monochromator. In order to maximize/tune the pulse intensity downstream of the monochromator, one therefore needs to adjust the averaged electron beam energy but also the details of the independent electron bunch energy within each pulse pair so that the corresponding X-ray averaged energy matches that of the monochromator for each pulse within the pulse pairs. There is unfortunately not currently the possibility to access immediately and non-destructively the electron energy distribution of each X-ray pulse within a pulse pair, other than with the XTCAV. However, the diagnostics presented here allows the intensity of each monochromatic pulse within a pair (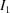, 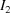) to be characterized parasitically as a function of the reduced electron mean energy 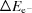.

This is illustrated in Figs. 4(*b*) and 4(*c*) ▸, which represent two-dimensional cumulative histograms of 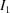 and 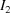 as a function of the reduced electron beam energy 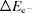. They correspond to the configuration (1) described in the previous section where a good agreement between the center of the average SASE spectrum for both pulses within a pair and the monochromator central energy is achieved. This is specifically highlighted by the alignment of the peak of the photon pulse intensity distributions of 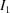 and 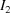 with that of the electron energy, as indicated by the dashed lines. One also consistently observes that the intensity distributions of 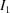 and 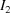 are nearly empty for pulses of almost zero intensity with a mean X-ray central energy, as described in Figs. 3(*a*) and 3(*c*) ▸.

In contrast, Figs. 4(*d*)–4(*f*) ▸ display the same quantities as Figs. 4(*a*)–4(*c*) ▸ but for configuration (2), which was described in the previous section, and Figs. 3(*b*) and 3(*d*) ▸. It highlights a different scenario where for only one of the two pulses (*i.e.* 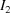) the central photon energy is aligned with that of the monochromator which is fixed by design to 8.2 keV. In this case, the mean of the 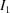 intensity distribution is offset by 2 MeV compared with that of 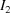, which translates into a difference in X-ray photon energy of about 2 eV. The differences described in the previous section can also be observed here. This is especially the case for the first pulse whose distribution is not symmetric around 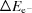 = 0 and also for the second pulse for which the most likely intensities are zero and centered to 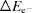 = 1 MeV. It also explains the reason behind the difference in correlation between 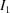 and 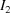 shown in Figs. 3(*b*) and 3(*d*) ▸.

This demonstrates the benefit of characterizing non-destructively the behavior of both 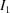 and 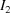 in relation to one of the parameters from the electron bunch. The immediate and online access to this information during the experiment undoubtedly provides a path to guide the optimization of the machine configuration for the nanosecond double-bunch operation mode that can be tailored to specific experimental needs.

## 4. Conclusion   

A compact transmissive pair intensity diagnostic for the LCLS nanosecond double-bunch mode was developed and tested. It provides a beamline monitor that has the unique capability of distinguishing two pulses at time intervals as short as 0.7 ns. As a shot-to-shot diagnostic, it serves as an indispensable tool for 2P-XSVS experiments where measuring the pulse intensities of two pulses is of key importance to data classification, correction and interpretation. It is equally important for X-ray pump/X-ray probe experiments, for which a clear characterization of the intensity of each pulse within a pair will allow the data to be binned to reconstruct a pump-fluence-dependence analysis. When combined with other beamline diagnostic measurements such as the electron beam energy, it shines new light on the details of the double-bunch mode and provides valuable guidance to the fine-tuning of the accelerator and lasing process in order to optimize the photon beam characteristics to specific experimental requirements.
